# Supplementary material for: Behavioral insights into audience satisfaction: analyzing emotional and cognitive factors in K-culture dance engagement
Source: PLoS One. 2025 Dec 12;20(12):e0337527. doi: 10.1371/journal.pone.0337527 (PMC12700408; doi:10.1371/journal.pone.0337527)
Supplement: S1 File — (DOCX) [file pone.0337527.s001.docx]

**Appendix A: Principal Component Analysis (PCA) results.**

**(1) Personality Traits Analysis**

Table A1. KMO and Bartlett’s Test (Personality)

| **Test** | **Value** |
| --- | --- |
| Kaiser-Meyer-Olkin Measure of Sampling Adequacy | 0.716 |
| Bartlett’s Test of Sphericity—Approx. Chi-Square | 321.503 |
| df | 15 |
| Sig. | <0.001 |

Table A2. Total Variance Explained (Personality).

| **Component** | **Initial Eigenvalues** |  | **Extraction Sums of Squared Loadings** |  | **Rotation Sums of Squared Loadings** |  |  |
| --- | --- | --- | --- | --- | --- | --- | --- |
|  | Total | % of Variance | Cumulative % | Total | % of Variance | Cumulative % | Total |
| 1 | 2.511 | 41.85 | 41.85 | 2.511 | 41.85 | 41.85 | 2.178 |
| 2 | 1.046 | 17.44 | 59.28 | 1.046 | 17.44 | 17.44 | 1.379 |

Table A3. Component Matrix After Rotation (Personality)

| **Indicators** | **Comp1** | **Comp2** |
| --- | --- | --- |
| X1 | –0.370 | 0.644 |
| X2 | 0.768 | –0.192 |
| X3 | 0.156 | 0.782 |
| X4 | 0.725 | –0.102 |
| X5 | –0.553 | 0.552 |
| X6 | 0.771 | 0.039 |

Principal Component Equation (Personality):

PC1=−0.0064X1+0.2709X2+0.2398X3+0.2735X4−0.1019X5+0.3241X6

PC1=−0.0064*X*1+0.2709*X*2+0.2398*X*3+0.2735*X*4−0.1019*X*5+0.3241*X*6 (II-1)

**(2) Emotional response analysis**

Table A4. KMO and Bartlett’s Test (Emotion)

| **Test** | **Value** |
| --- | --- |
| Kaiser-Meyer-Olkin Measure of Sampling Adequacy | 0.625 |
| Bartlett’s Test of Sphericity—Approx. Chi-Square | 76.916 |
| df | 10 |
| Sig. | <0.001 |

Table A5. Total Variance Explained (Emotion)

| **Component** | **Initial Eigenvalues** |  | **Extraction Sums of Squared Loadings** |  | **Rotation Sums of Squared Loadings** |  |  |
| --- | --- | --- | --- | --- | --- | --- | --- |
|  | Total | % of Variance | Cumulative % | Total | % of Variance | Cumulative % | Total |
| 1 | 1.666 | 33.32 | 33.32 | 1.666 | 33.32 | 33.32 | 1.545 |
| 2 | 1.052 | 21.04 | 54.36 | 1.052 | 21.04 | 54.36 | 1.173 |

Table A6. Component Matrix After Rotation (Emotion)

| **Indicators** | **Comp1** | **Comp2** |
| --- | --- | --- |
| X1 | 0.092 | 0.809 |
| X2 | –0.724 | 0.182 |
| X3 | 0.657 | 0.120 |
| X4 | 0.725 | –0.150 |
| X5 | –0.235 | 0.669 |

Principal Component Equation (Emotion):

PC2=0.3157X1−0.7512X2+0.7571X3+0.7608X4−0.0824X5

PC2=0.3157*X*1−0.7512*X*2+0.7571*X*3+0.7608*X*4−0.0824*X*5 (II-2)

**(3) Environmental quality analysis**

Table A7. KMO and Bartlett’s Test (Environment)

| **Test** | **Value** |
| --- | --- |
| Kaiser-Meyer-Olkin Measure of Sampling Adequacy | 0.857 |
| Bartlett’s Test of Sphericity—Approx. Chi-Square | 806.717 |
| df | 10 |
| Sig. | <0.001 |

Table A8. Total Variance Explained (Environment)

| **Component** | **Initial Eigenvalues** |  | **Extraction Sums of Squared Loadings** |  | **Rotation Sums of Squared Loadings** |  |  |
| --- | --- | --- | --- | --- | --- | --- | --- |
|  | Total | % of Variance | Cumulative % | Total | % of Variance | Cumulative % | Total |
| 1 | 3.549 | 70.99 | 70.99 | 3.549 | 70.99 | 70.99 | 2.297 |
| 2 | 0.486 | 9.72 | 80.71 | 0.486 | 9.72 | 9.72 | 1.738 |

Table A9. Component Matrix After Rotation (Environment)

| **Indicators** | **Comp1** | **Comp2** |
| --- | --- | --- |
| X1 | 0.276 | 0.930 |
| X2 | 0.697 | 0.557 |
| X3 | 0.728 | 0.496 |
| X4 | 0.920 | 0.201 |
| X5 | 0.599 | 0.526 |

Principal Component Equation (Environment):

PC3=0.1966X1+0.2118X2+0.2075X3+0.1946X4+0.1895X5

PC3=0.1966*X*1+0.2118*X*2+0.2075*X*3+0.1946*X*4+0.1895*X*5 (II-3)

**(4) Overall satisfaction analysis**

Table A10. KMO and Bartlett’s Test (Satisfaction)

| **Test** | **Value** |
| --- | --- |
| Kaiser-Meyer-Olkin Measure of Sampling Adequacy | 0.835 |
| Bartlett’s Test of Sphericity—Approx. Chi-Square | 689.502 |
| df | 6 |
| Sig. | <0.001 |

Table A11. Total Variance Explained (Satisfaction)

| **Component** | **Initial Eigenvalues** |  | **Extraction Sums of Squared Loadings** |  | **Rotation Sums of Squared Loadings** |  |  |
| --- | --- | --- | --- | --- | --- | --- | --- |
|  | Total | % of Variance | Cumulative % | Total | % of Variance | Cumulative % | Total |
| 1 | 3.104 | 77.61 | 77.61 | 3.104 | 77.61 | 77.61 | 1.940 |
| 2 | 0.383 | 9.58 | 87.19 | 0.383 | 9.58 | 87.19 | 1.547 |

Table A12. Component matrix after rotation (Satisfaction).

| **Indicators** | **Comp1** | **Comp2** |
| --- | --- | --- |
| X1 | 0.890 | 0.318 |
| X2 | 0.341 | 0.914 |
| X3 | 0.804 | 0.433 |
| X4 | 0.620 | 0.651 |

Principal Component Equation (Satisfaction):

PC4=0.2474X1+0.2465X2+0.2514X3+0.2546X4

PC4=0.2474*X*1+0.2465*X*2+0.2514*X*3+0.2546*X*4 (II-4)
