# Supplementary material for: Behavioral insights into audience satisfaction: analyzing emotional and cognitive factors in K-culture dance engagement
Source: PLoS One. 2025 Dec 12;20(12):e0337527. doi: 10.1371/journal.pone.0337527 (PMC12700408; doi:10.1371/journal.pone.0337527)
Supplement: S2 File — (DOCX) [file pone.0337527.s002.docx]

**Human Participants Research Checklist**

***Complete the following if your study involved human participants or human participants’ data. These questions should be addressed for prospective and retrospective studies.***

1. Did you obtain ethics approval for this study?
   - If yes, please upload (file type “Other”) all the approval documents you received from your ethics committee to cover the entire range of the study period (i.e. the original approval document and any extension documents). Where ethics approval was obtained from more than one study location, please provide approval document(s) from all of the sites. If the original document is in another language, please also provide an English translation.

___ Uploaded _v__ N/A

- - If you did not obtain ethical approval, please explain why this was not required below.

Ethics approval was not required because the study involved the collection of anonymous survey responses from dance performance audience members. Participants were randomly selected and provided informed consent. No identifiable personal information was collected, and the data do not allow identification of individual participants. According to institutional and national guidelines, research involving fully anonymous, non-sensitive survey data collected from adults does not require ethics committee approval.

1. If you prospectively recruited human participants for the study – for example, you conducted a clinical trial, distributed questionnaires, or obtained tissues, data or samples for the purposes of this study, please report in the Methods:
   1. the day, month and year of the **start and end** of the recruitment period for this study.
   2. whether participants provided informed consent, and if so, what type was obtained (for instance, written or verbal, and if verbal, how it was documented and witnessed). If your study included minors, state whether you obtained consent from parents or guardians. If the need for consent was waived by the ethics committee, please include this information.

Please state the line number(s) in the Methods where this is reported ______

_v__ Completed ___ N/A

In the Methods section of the manuscript, we have reported that participants were randomly selected audience members after a dance performance. Participation was voluntary and responses were collected anonymously through a structured questionnaire. No personal identifiers or sensitive information were collected.

1. If you are reporting a retrospective study of, for example, medical records, archived samples, survey data, please report in the Methods section:
2. the day, month and year when the data were accessed for research purposes
3. whether authors had access to information that could identify individual participants during or after data collection

Please state the line number(s) in the Methods where this is reported ______

__v_ Completed ___ N/A

Yes. All participants were informed about the nature and purpose of the survey, and participation was entirely voluntary. Completion of the anonymous questionnaire was considered to indicate informed consent.
