# Supplementary material for: Behavioral insights into audience satisfaction: analyzing emotional and cognitive factors in K-culture dance engagement
Source: PLoS One. 2025 Dec 12;20(12):e0337527. doi: 10.1371/journal.pone.0337527 (PMC12700408; doi:10.1371/journal.pone.0337527)
Supplement: Attachment — Submitted filename: comments.docx [file pone.0337527.s003.docx]

# Reviewer Report

This report provides a critical review of the manuscript entitled “Behavioral Insights into Audience Satisfaction: Analyzing Emotional and Cognitive Factors in K-Culture Dance Engagement.” The review identifies strengths, weaknesses, and areas for improvement.

**Comments**

1. Title/Abstract: The title is specific. The abstract is however descriptive and does not contain explicit statement of theoretical contribution. The authors ought to put emphasis on the contribution of the study to theory and practice in the study of audience satisfaction.

2. Introduction: The statement of the problem is to be expressed in a clearer manner and then be succeeded by a number of research questions or hypotheses.

3. Literature Review: The literature review is extensive and informative but in some cases it reminds of a cultural essay more than a specialized synthesis. Certain areas (e.g. digital engagement, AR/Tik Tok) are not obviously aligned with the empirical models. The review needs to be minimal in terms of supporting the variables of the study.

4. Theoretical Framework: Despite the mentioning of the factors, emotional, cognitive and environmental, the paper lacks a clearly defined anchoring theory. It should provide a conceptual model which is connected to a particular framework like Expectancy-Disconfirmation or Social Cognitive Theory.

5. Methodology: Data collection is outlined in detail but the sampling method used (one performance event) constrains representativeness. Also, there is no clarity in the validation of newly developed items. Pilot testing, reliability and validity tests should be reported in the paper.

6. Discussion: The discussion paraphrases results, but does not critically reflect. Other reasons like culture norm or social desirability bias ought to be addressed. There is also no clear limitations section present.

7. Conclusion: The conclusion cannot be described as adequate focus on theoretical progress. The authors ought to explain the ways in which their results can be generalized to the audience satisfaction theory.

8. References and Formatting: The reference list is very long however it contains secondary references, repeated references and broken links. The authors are to give the preference to peer-reviewed sources and adhere to APA formatting.
