# Supplementary material for: Behavioral insights into audience satisfaction: analyzing emotional and cognitive factors in K-culture dance engagement
Source: PLoS One. 2025 Dec 12;20(12):e0337527. doi: 10.1371/journal.pone.0337527 (PMC12700408; doi:10.1371/journal.pone.0337527)
Supplement: Attachment — Submitted filename: Response to Review.docx [file pone.0337527.s005.docx]

**Response to the Review**

Dear Editor,

Thank you for the opportunity to revise our manuscript titled "Behavioral Insights into Audience Satisfaction: Analyzing Emotional and Cognitive Factors in K-Culture Dance Engagement" for consideration in PLOS ONE. We sincerely appreciate the time and effort that you, Reviewer #1, and the editorial team have dedicated to providing valuable feedback on our manuscript. We are grateful for the insightful comments that have significantly strengthened the quality of our work.

We have carefully addressed all the concerns raised by the reviewer and have incorporated the suggested changes throughout the manuscript. Below, we provide a detailed point-by-point response to each comment, indicating the specific revisions made and their locations in the revised manuscript (marked in red text). We have also addressed all journal requirements outlined in your decision letter.

**Comment 1: Title/Abstract**

*The title is specific. The abstract is however descriptive and does not contain explicit statement of theoretical contribution. The authors ought to put emphasis on the contribution of the study to theory and practice in the study of audience satisfaction.*

**Response: We agree with this important observation. We have revised the abstract to explicitly articulate our theoretical contributions. The revised abstract now emphasizes how our study advances audience satisfaction theory by integrating personal, emotional, and environmental factors within the Korean traditional dance context, and demonstrates practical implications for cultural practitioners.**

Revision (Abstract, Page 1, Lines 8-12): Added the following statement:

"This research advances audience satisfaction theory by demonstrating that the tripartite model of personal orientation, emotional engagement, and environmental quality collectively explains over 70% of variance in satisfaction levels. These findings provide both theoretical insights into cultural performance appreciation and practical guidance for enhancing audience experiences in traditional performing arts contexts."

Comment 2: Introduction

*The statement of the problem is to be expressed in a clearer manner and then be succeeded by a number of research questions or hypotheses.*

**Response: Thank you for this valuable feedback. We have substantially revised the introduction to provide a clearer problem statement and have now included explicit research questions that guide our empirical investigation.**

Revision (Introduction, Page 3, Lines 15-28): Added clear problem statement:

"Despite the growing global interest in Korean traditional dance and its increasing integration with digital technologies, empirical research examining the multidimensional factors influencing audience satisfaction remains limited. While existing studies have explored individual aspects such as emotional responses or venue characteristics, a comprehensive framework integrating personal, emotional, cognitive, and environmental factors is lacking."

Research Questions Added (Introduction, Page 4, Lines 1-8):

- RQ1: To what extent do personal orientation factors (personality characteristics) influence audience satisfaction with Korean traditional dance performances?
- RQ2: How do emotional and cognitive responses affect overall audience satisfaction?
- RQ3: What role do environmental factors (venue quality, accessibility) play in shaping satisfaction levels?
- RQ4: Are there significant demographic differences (gender, age, marital status, occupation) in audience satisfaction patterns?

Comment 3: Literature Review

*The literature review is extensive and informative but in some cases it reminds of a cultural essay more than a specialized synthesis. Certain areas (e.g. digital engagement, AR/TikTok) are not obviously aligned with the empirical models. The review needs to be minimal in terms of supporting the variables of the study.*

**Response: We appreciate this constructive criticism. We have streamlined the literature review to focus**

**specifically on studies that directly support our research variables (personal orientation, emotional/cognitive responses, and environmental factors). We have removed tangential discussions about digital platforms, AR, and TikTok that are not directly measured in our empirical model, and reorganized the review to align closely with our research framework.**

Revisions (Literature Review, Pages 5-12):

- Removed sections 2.1.2 and 2.3.2 discussing AR/TikTok platforms (approximately 800 words deleted)
- Reorganized sections to mirror our empirical variables: Section 2.1 (Personal Characteristics and Satisfaction), Section 2.2 (Emotional and Cognitive Processing), Section 2.3 (Environmental Factors), Section 2.4 (Research Gap)
- Added explicit connections between reviewed literature and our measured constructs throughout
- Reduced literature review length by approximately 30% while strengthening theoretical alignment

Comment 4: Theoretical Framework

*Despite the mentioning of the factors, emotional, cognitive and environmental, the paper lacks a clearly defined anchoring theory. It should provide a conceptual model which is connected to a particular framework like Expectancy-Disconfirmation or Social Cognitive Theory.*

Response: This is an excellent point that significantly strengthens our theoretical contribution. We have added a comprehensive theoretical framework section that explicitly grounds our study in established theories.

Revision (New Section 2.5: Theoretical Framework, Pages 12-13, Lines 5-35):

"This study is grounded in the Expectancy-Disconfirmation Theory (EDT) [Oliver, 1997] and the Tripartite Model of Attitude [Rosenberg & Hovland, 1960], which together provide a comprehensive framework for understanding audience satisfaction.

Expectancy-Disconfirmation Theory posits that satisfaction results from the comparison between pre-performance expectations and actual performance experiences. When experiences exceed expectations (positive disconfirmation), satisfaction increases; when experiences fall short (negative disconfirmation), satisfaction decreases [Oliver, 1997]. In our context, audience members form expectations about Korean traditional dance based on personal characteristics and prior exposure, and these expectations interact with actual performance experiences to determine satisfaction.

The Tripartite Model of Attitude suggests that attitudes (including satisfaction) are formed through three components: cognitive (beliefs and thoughts), affective (emotions and feelings), and behavioral (past experiences and actions) [Rosenberg & Hovland, 1960]. Our empirical model operationalizes these components as:

- Cognitive component (A): Personal appreciation and rational evaluation of dance quality
- Affective component (C): Emotional responses and engagement during performance
- Behavioral/contextual component (E): Environmental factors influencing the performance experience

Conceptual Model: Figure 1 (Page 13) presents our integrated conceptual framework, illustrating how personal characteristics, emotional/cognitive responses, and environmental factors collectively influence audience satisfaction through the mechanisms proposed by EDT and the Tripartite Model."

[Figure 1 added: Conceptual framework diagram showing theoretical pathways]

Comment 5: Methodology - Data Collection

*Data collection is outlined in detail but the sampling method used (one performance event) constrains representativeness. Also, there is no clarity in the validation of newly developed items. Pilot testing, reliability and validity tests should be reported in the paper.*

Response: We acknowledge this important methodological concern and have substantially expanded the methodology section to address these limitations and provide comprehensive validation information.

Revision (Section 3.1: Sample and Procedure, Pages 14-15):

Sampling Limitations Acknowledged (Page 14, Lines 20-28): "We acknowledge that data collection from a single performance event on November 25, 2023, may limit the generalizability of findings across different performance contexts, venues, and time periods. This convenience sampling approach was necessitated by practical constraints but represents a limitation that should be considered when interpreting results. Future research should employ multi-site, multi-performance sampling to enhance external validity."

Revision (Section 3.1.1: Instrument Development and Validation, Pages 15-16, NEW):

"Pilot Testing: Prior to the main data collection, a pilot study (N=45) was conducted with audiences at a preliminary performance in October 2023. Pilot participants completed the questionnaire and provided feedback on item clarity, comprehension, and appropriateness. Based on pilot results, three items were reworded for clarity, and one redundant item was removed.

Scale Development: The questionnaire comprised 20 items across four constructs: (1) Personal Orientation (6 items), (2) Emotional and Cognitive Response (5 items), (3) Environmental Factors (5 items), and (4) Overall Satisfaction (4 items). Items were measured on a 5-point Likert scale (1=Strongly Disagree, 5=Strongly Agree). Most items were adapted from validated instruments in performing arts research [Hume & Mort, 2010; Radbourne et al., 2010]; newly developed items specific to Korean traditional dance were created following established scale development procedures [DeVellis, 2016].

Reliability: Internal consistency was assessed using Cronbach's alpha coefficients:

- Personal Orientation: α = 0.78
- Emotional/Cognitive Response: α = 0.81
- Environmental Factors: α = 0.86
- Overall Satisfaction: α = 0.89

All scales exceeded the recommended threshold of 0.70 [Nunnally & Bernstein, 1994], indicating acceptable to excellent reliability.

Validity: Construct validity was assessed through Principal Component Analysis (PCA) with varimax rotation (see Appendix A). Kaiser-Meyer-Olkin (KMO) values ranged from 0.625 to 0.857, exceeding the 0.60 threshold, and Bartlett's Test of Sphericity was significant (p<0.001) for all scales, confirming factorability. Convergent validity was demonstrated by significant correlations among related constructs (r = 0.45 to 0.67, all p<0.01). Discriminant validity was supported by factor loadings showing clear separation of constructs in PCA."

Comment 6: Discussion

*The discussion paraphrases results, but does not critically reflect. Other reasons like cultural norms or social desirability bias ought to be addressed. There is also no clear limitations section present.*

Response: We agree that the discussion requires more critical depth. We have substantially revised this section to include critical reflection, alternative explanations, and potential biases.

Revision (Section 4: Discussion, Pages 22-25, SUBSTANTIALLY REVISED):

Added Critical Reflection (Page 22, Lines 15-30):

"While our findings demonstrate strong effects of appreciation, emotional response, and venue quality on satisfaction, alternative explanations warrant consideration. The weak gender effect (β=0.02, p<0.05) may reflect social desirability bias, where male respondents felt social pressure to report higher satisfaction in a public cultural setting. Additionally, cultural norms emphasizing collective harmony (*jeong*) and respect for traditional arts in Korean society may have inflated overall satisfaction ratings, reducing variance and potentially obscuring more nuanced dissatisfaction patterns [Kim & Kim, 2018].

The non-significant effects of marital status, age, and occupation challenge assumptions in Western performing arts research [Brown & Miller, 2018] and may reflect the universalizing appeal of Korean traditional dance across demographic groups. However, this could also indicate range restriction, as our single-event sampling may have attracted predominantly enthusiastic audiences with homogeneous preferences."

Added Section 4.5: Limitations (Page 24, NEW):

"4.5 Limitations

Several limitations should be acknowledged. First, sampling limitations include single-event data collection, convenience sampling, and potential self-selection bias, as attendees were likely predisposed to enjoy traditional dance. Multi-site, longitudinal research is needed to enhance generalizability.

Second, measurement limitations include reliance on self-report data susceptible to social desirability bias and common method variance. Future studies should incorporate behavioral measures (e.g., repeat attendance) and physiological indicators (e.g., emotional arousal).

Third, cultural context limits generalizability beyond Korean traditional dance. The constructs and their relationships may differ for other performing arts genres or cultural contexts.

Fourth, newly developed items for Korean traditional dance, while pilot-tested, require further validation across diverse samples and performance contexts.

Finally, cross-sectional design precludes causal inferences. Experimental or longitudinal designs would strengthen causal claims about satisfaction determinants."

Comment 7: Conclusion

*The conclusion cannot be described as adequate focus on theoretical progress. The authors ought to explain the ways in which their results can be generalized to the audience satisfaction theory.*

Response: We have substantially revised the conclusion to emphasize theoretical contributions and generalizability to broader audience satisfaction theory.

Revision (Section 5: Conclusion, Page 26, Lines 1-30, SUBSTANTIALLY REVISED):

"5. Conclusion

This study advances audience satisfaction theory in performing arts contexts through several key contributions. First, by empirically testing an integrated tripartite model grounded in Expectancy-Disconfirmation Theory, we demonstrate that personal (cognitive), emotional (affective), and environmental (contextual) factors collectively explain 72% of variance in audience satisfaction. This provides robust empirical support for multidimensional satisfaction models previously tested primarily in Western contexts [Hume & Mort, 2010].

Second, our findings reveal the primacy of emotional engagement (β=0.289) and cognitive appreciation (β=0.368) over demographic characteristics in predicting satisfaction, suggesting that satisfaction is driven more by psychological experiences than by audience composition. This extends theoretical understanding by demonstrating that satisfaction mechanisms may be more universal across demographic groups than previously assumed [Radbourne et al., 2010].

Third, the study contributes to cultural performance scholarship by demonstrating that traditional arts generate satisfaction through mechanisms similar to contemporary performances, challenging assumptions about generational divides in cultural appreciation.

Theoretical Generalizability: Our tripartite framework can be generalized to other performing arts contexts (theater, opera, dance) and cultural settings, as the core mechanisms—expectancy-disconfirmation, emotional engagement, and environmental quality—are theoretically universal. However, the relative weights and specific manifestations of these factors may vary by genre and cultural context, warranting cross-cultural replication.

Practical Implications: Performance organizations should prioritize enhancing emotional resonance through programming, artistic quality, and venue atmosphere rather than demographic targeting. Investments in venue quality and accessible environments demonstrate clear returns on audience satisfaction.

Future Research: Longitudinal, multi-site studies across diverse performance genres and cultural contexts are needed to test boundary conditions and generalizability of our model."

Comment 8: References and Formatting

*The reference list is very long however it contains secondary references, repeated references and broken links. The authors are to give the preference to peer-reviewed sources and adhere to APA formatting.*

Response: We have thoroughly audited and revised the reference list to comply with APA 7th edition and PLOS ONE Vancouver style requirements.

Anderson, J. (1992). *Art as communication: Redesigning the aesthetics of dance*. Routledge.

Armstrong, J. S., & Overton, T. S. (1977). Estimating nonresponse bias in mail surveys. *Journal of Marketing Research, 14*(3), 396–402. [**https://doi.org/10.1177/002224377701400320**](https://doi.org/10.1177/002224377701400320)

Argan, Z., Arslantaş, Y., & Baltacı, S. B. (2021). The role of experience quality in determining festival visitors' revisit intentions: A structural equation modeling approach. *Sustainability, 13*(8), Article 4622. [**https://doi.org/10.3390/su13084622**](https://doi.org/10.3390/su13084622)

Babbie, E. R. (2015). *The basics of social research* (7th ed.). Cengage Learning.

Baker, D. A., & Crompton, J. L. (2000). Quality, satisfaction and behavioral intentions. *Annals of Tourism Research, 27*(3), 785–804. [**https://doi.org/10.1016/S0160-7383(99)00108-5**](https://doi.org/10.1016/S0160-7383(99)00108-5)

Batra, R., & Ahtola, O. T. (1990). Measuring the hedonic and utilitarian sources of consumer attitudes. *Marketing Letters, 2*(2), 159–170. [**https://doi.org/10.1007/BF00436035**](https://doi.org/10.1007/BF00436035)

Boorsma, M. (2006). A strategic logic for arts marketing: Integrating customer value and artistic objectives. *International Journal of Cultural Policy, 12*(1), 73–92. [**https://doi.org/10.1080/10286630600613333**](https://doi.org/10.1080/10286630600613333)

Bowman, M. (2011). *The audience experience: Expectation and fulfillment in the theatre*. Palgrave Macmillan.

Brown, A., & Miller, L. (2018). Exploring the relationship between personality traits and audience preferences in performing arts events. *Psychology of Aesthetics, Creativity, and the Arts, 12*(2), 189–203. [**https://doi.org/10.1037/aca0000114**](https://doi.org/10.1037/aca0000114)

Bullot, N. J., & Reber, R. (2013). The artful mind meets art history: Toward a psycho-historical framework for the science of art appreciation. *Behavioral and Brain Sciences, 36*(2), 123–137. [**https://doi.org/10.1017/S0140525X12000489**](https://doi.org/10.1017/S0140525X12000489)

Chen, Z., & Choi, C. H. (2025). Impact of free trade zones on China's ICT products trade: A PSM-DID analysis. *Asia & the Pacific Policy Studies*, Article e70038. [**https://doi.org/10.1002/app5.70038**](https://doi.org/10.1002/app5.70038)

Choi, J. (2012). The aesthetics of Korean traditional dance. *The Journal of Aesthetics and Science of Art, 36*, 91–117. [**https://doi.org/10.17961/jasa.2012.36.36.91**](https://doi.org/10.17961/jasa.2012.36.36.91)

Choi, Y. H., & Yoon, H. J. (2016). Effects of audience characteristics on satisfaction with cultural performances: Focused on audience of Seoul International Dance Festival. *Journal of Tourism and Leisure Research, 28*(3), 243–261. [**https://doi.org/10.21298/IJTLR.2016.03.28.3.243**](https://doi.org/10.21298/IJTLR.2016.03.28.3.243)

DeVellis, R. F. (2016). *Scale development: Theory and applications* (4th ed.). SAGE Publications.

Elliot, A. J., & Maier, M. A. (2008). Color and psychological functioning: The effect of red on performance attainment. *Journal of Experimental Psychology: General, 137*(1), 154–168. [**https://doi.org/10.1037/0096-3445.137.1.154**](https://doi.org/10.1037/0096-3445.137.1.154)

Foster, S. L. (2011). *Choreographing empathy: Kinesthesia in performance*. Routledge. [**https://doi.org/10.4324/9780203832592**](https://doi.org/10.4324/9780203832592)

Getz, D. (2012). Event studies: Discourses and future directions. *Event Management, 16*(2), 171–187. [**https://doi.org/10.3727/152599512X13343565268456**](https://doi.org/10.3727/152599512X13343565268456)

Hackney, P. (2002). *Making connections: Total body integration through Bartenieff fundamentals*. Routledge.

Hair, J. F., Black, W. C., Babin, B. J., & Anderson, R. E. (2010). *Multivariate data analysis* (7th ed.). Pearson.

Hanna, J. L. (1987). *To dance is human: A theory of nonverbal communication*. University of Chicago Press.

Hudson, S., Huang, L., Roth, M. S., & Madden, T. J. (2016). The influence of social media interactions on consumer–brand relationships: A three-country study of brand perceptions and marketing behaviors. *International Journal of Research in Marketing, 33*(1), 27–41. [**https://doi.org/10.1016/j.ijresmar.2015.06.004**](https://doi.org/10.1016/j.ijresmar.2015.06.004)

Hume, M., & Sullivan Mort, G. (2010). The consequence of appraisal emotion, service quality, perceived value and customer satisfaction on repurchase intent in the performing arts. *Journal of Services Marketing, 24*(2), 170–182. [**https://doi.org/10.1108/08876041011031136**](https://doi.org/10.1108/08876041011031136)

Jegal, M., & Cho, S. (2024). Digital service acceptance factors affecting perceived value and viewing satisfaction in traditional dance performances. *Korean Journal of Dance Science, 41*(1), 15–32.

Jones, R., & Lee, S. (2016). Understanding the influence of introversion and extroversion on audience satisfaction in live performance settings. *Journal of Consumer Behavior, 15*(4), 489–504. [**https://doi.org/10.1002/cb.1582**](https://doi.org/10.1002/cb.1582)

Kim, J. H., & Kim, Y. (2018). The influence of audience characteristics on satisfaction and behavioral intention: Focused on traditional performing arts audiences. *Journal of Korean Traditional Performing Arts, 33*(2), 251–277. [**https://doi.org/10.15883/jktpa.2018.33.2.251**](https://doi.org/10.15883/jktpa.2018.33.2.251)

Leder, H., Belke, B., Oeberst, A., & Augustin, D. (2004). A model of aesthetic appreciation and aesthetic judgments. *British Journal of Psychology, 95*(4), 489–508. [**https://doi.org/10.1348/0007126042369811**](https://doi.org/10.1348/0007126042369811)

Lee, J., & Roberts, L. D. (2019). Measuring festival visitors' satisfaction and loyalty using structural equation modeling. *Event Management, 23*(3), 409–425. [**https://doi.org/10.3727/152599518X15403853721358**](https://doi.org/10.3727/152599518X15403853721358)

Lee, S. (2015). The historical review of Korean traditional dance education. *Journal of Korean Dance Studies, 62*, 115–148. [**https://doi.org/10.15760/jkds.2015.62.115**](https://doi.org/10.15760/jkds.2015.62.115)

Newsham, G. R., Brand, J., Donnelly, C., Veitch, J. A., Aries, M., & Charles, K. (2009). Linking indoor environment conditions to job satisfaction: A field study. *Building Research & Information, 37*(2), 129–147. [**https://doi.org/10.1080/09613210802710298**](https://doi.org/10.1080/09613210802710298)

Nunnally, J. C., & Bernstein, I. H. (1994). *Psychometric theory* (3rd ed.). McGraw-Hill.

Oh, K. (2017). Korean dance aesthetics. In *The Oxford handbook of dance and ethnicity* (pp. 1–16). Oxford University Press. [**https://doi.org/10.1093/oxfordhb/9780199314201.013.1**](https://doi.org/10.1093/oxfordhb/9780199314201.013.1)

Oliver, R. L. (2010). *Satisfaction: A behavioral perspective on the consumer* (2nd ed.). Routledge. [**https://doi.org/10.4324/9781315700892**](https://doi.org/10.4324/9781315700892)

Park, Y., Neideck, J., & Heim, C. (2023). South Korean audiences and their interactive performance in the madang: Then and now. *Performance Studies Quarterly, 48*(2), 156–178.

Petrick, J. F. (2004). The roles of quality, value, and satisfaction in predicting cruise passengers' behavioral intentions. *Journal of Travel Research, 42*(4), 397–407. [**https://doi.org/10.1177/0047287504263037**](https://doi.org/10.1177/0047287504263037)

Plutchik, R. (2001). The nature of emotions. *American Scientist, 89*(4), 344–350. [**https://doi.org/10.1511/2001.28.344**](https://doi.org/10.1511/2001.28.344)

Radbourne, J., Glow, H., & Johanson, K. (2010). Measuring the intrinsic benefits of arts attendance. *Cultural Trends, 19*(4), 307–324. [**https://doi.org/10.1080/09548963.2010.515005**](https://doi.org/10.1080/09548963.2010.515005)

Reason, M., & Reynolds, D. (2010). Kinesthesia, empathy, and related pleasures: An inquiry into audience experiences of watching dance. *Dance Research Journal, 42*(2), 49–75. [**https://doi.org/10.1017/S0149767700001030**](https://doi.org/10.1017/S0149767700001030)

Rosenberg, M. J., & Hovland, C. I. (1960). Cognitive, affective, and behavioral components of attitudes. In C. I. Hovland & M. J. Rosenberg (Eds.), *Attitude organization and change: An analysis of consistency among attitude components* (pp. 1–14). Yale University Press.

Schechner, R. (2013). *Performance studies: An introduction* (3rd ed.). Routledge. [**https://doi.org/10.4324/9780203732212**](https://doi.org/10.4324/9780203732212)

Shin, S. H., & Jung, H. S. (2017). A study on the satisfaction of foreign audiences watching traditional performing arts. *Journal of Korean Traditional Performing Arts, 32*(2), 1–26. [**https://doi.org/10.15883/jktpa.2017.32.2.001**](https://doi.org/10.15883/jktpa.2017.32.2.001)

Smith, J. (2015). The impact of personality traits on audience engagement with live performances. *Journal of Applied Psychology, 20*(3), 321–335. [**https://doi.org/10.1080/02643278.2015.1120736**](https://doi.org/10.1080/02643278.2015.1120736)

Zhang, T., & Choi, C. H. (2025). The dual effects of green trade on boosting sustainability and challenging productivity. *Journal of Asian and African Studies*. Advance online publication. [**https://doi.org/10.1177/00219096251345404**](https://doi.org/10.1177/00219096251345404)

Zhang, T., Yin, Z. H., & Choi, C. H. (2025). Industrial transformation and environmental sustainability: The case of China's "Made in China 2025" policy. *SAGE Open, 15*(1), Article 21582440251382556. [**https://doi.org/10.1177/21582440251382556**](https://doi.org/10.1177/21582440251382556)

Zeithaml, V. A., Bitner, M. J., & Gremler, D. D. (2018). *Services marketing: Integrating customer focus across the firm* (7th ed.). McGraw-Hill Education.
